# Medical education during the COVID-19 pandemic: perspectives from UK trainees

**DOI:** 10.1136/postgradmedj-2020-137970

**Published:** 2020-05-05

**Authors:** Jason Yuen, Fangyi Xie

**Affiliations:** South West Neurosurgery Centre, Derriford Hospital, Plymouth, UK; Department of Dermatology, Royal Devon and Exeter Hospital, Exeter, UK

**Keywords:** medical education & training, education & training (see medical education & training)

The current COVID-19 pandemic has led to unprecedented disruptions to medical training. In this article, we will share two UK trainees’ perspectives on this.

Widespread interruptions to medical education are seen throughout history. At times of major conflicts, the quality of training suffers as a result.^1  2^ For example, during the blitz, students and newly qualified interns were distributed to areas of need.^2^ Also during World War II, certain American medical schools shortened their postgraduate degree programme from 4 years to 3 years to address doctor shortages.^3^ The UK government has adopted this to a degree, where final-year students were encouraged to start their Foundation Programme early.^4^

Despite the disruptions, there are always silver linings. After the two world wars, there have been radical reforms in the medical education system, leading to improvement of the curricula and intake,^5^ including an increase in women admissions to medical schools.^6^

The current pandemic is comparable to the other historical events, with major challenges to the National Health Service. Also, due to the drastic reduction in elective surgery and routine work, traditional learning opportunities have become scarcer. Many outpatient clinics have switched toward teleclinics and tele-triaging. Trainers have increasing demands on their time as they adapt to different ways of working. Even within a department, social distancing limits the availability of face-to-face contact with trainers. However, we now have more technical tools to enable remote learning, despite billions of the world population in lockdown.^7^

E-learning is not a new concept, with multiple platforms providing different learning materials, such as eBrain (http://www.ebrain.net) and e-learning for healthcare (https://www.e-lfh.org.uk). Nonetheless, recently we have experienced a rise in innovative trainers (including peer-to-peer trainers) who are creating new learning materials and/or holding videoconferences for learners worldwide, using software such as Zoom (https://zoom.us). Often these trainers have no prior experience with preparing e-learning materials.

There is evidence that videoconferences are non-inferior to face-to-face education.^8^ We have welcomed these, as they provide valuable learning opportunities from national and international experts, which trainees may otherwise miss out on. We hope, similar to the beneficial changes in curriculum we saw after the world wars, this trend will persist to benefit the coming generations of trainees post-pandemic, increasing the breadth of teaching resources available and reduce the time and cost of travelling for teaching.

Due to an increasing number of patients suffering from COVID-19, trainee doctors from other specialties have been redeployed to help manage these patients. This certainly provides challenges to those who may need to work in areas outside their comfort zone, but it also provides training opportunities and revision in the management of acutely unwell patients. Some may argue the knowledge would not be useful in the future, but one may also argue that even a neurosurgeon or dermatologist would be expected to recognise and treat a patient having a respiratory decompensation until help arrives.

Moreover, with the increasing amount of literature and news on COVID-19, there are ample opportunities to practise evidence-based medicine. The medical mind naturally engages to critically analyse the published materials against what is reported in the news to discern ‘fake news’, so we can inform our patients with the correct information.

Perhaps the key learning to be taken out of the crisis is not the clinical facts but the softer skills of leadership, innovation to adapt and team building. Clinicians are changing their pattern of working on a daily basis, and trainees may also be able to contribute their viewpoints to improve care. This is a great opportunity for trainees to learn excellent leadership skills from seniors at the trust, directorate and team level.

As the General Medical Council guidelines rightly point out, a doctor should ‘make the care of your patient your first concern’.^9^ We believe as a collective body of health professionals, we should prioritise the management of the COVID-19 outbreak before medical education in the current situation. However, it does not imply a complete halt of learning opportunities. In fact, both trainees and trainers need to start planning how we can facilitate trainees to continue their training pathway when it eventually returns to normal.

There are certain aspects of medical education that cannot be replaced by online resources. For surgical trainees, one of the biggest concerns is the lack of operations due to cancellation of elective cases. Although some guidelines have been published to try to clarify the assessment format and requirements,^10^ it has caused a lot of anxiety among trainees. More clarification on how the annual appraisals will be conducted is necessary.

In addition to just passing the appraisals, we also need ensure the trainees are clinically competent, and for those who are redeployed to other specialties that they do not become underskilled in their specialty once they return. There is uncertainty about whether training will need to be extended, and whether certificates of completion of training will need to be delayed.

We suggest there are three aspects that trainers can focus on during the outbreak (where possible) and more importantly when the outbreak subsides:

1. Preparation of revision materials for trainees who were preparing for their professional examinations.
2. Facilitating practical skills such as operations and clinical procedures (which will require patience and more patience), perhaps supplemented by simulation and technologies such as augmented or virtual reality.
3. Encourage trainees to reflect on what we have learned in this crisis and move forward.

Our main focus at the moment is patient care; however, we should not forget about the disruption in our medical education curriculum and its potential long-term impact. There are activities that both trainers and trainees can undertake in the current situation and afterwards to maximise the learning opportunities with the resources available. We also hope some of the changes improvised by our enthusiastic trainers in this crisis would persist in the future.
